# Methodologies of Primary HPV Testing Currently Applied for Cervical Cancer Screening

**DOI:** 10.3390/life10110290

**Published:** 2020-11-19

**Authors:** Andreas C. Chrysostomou, Leondios G. Kostrikis

**Affiliations:** Department of Biological Sciences, University of Cyprus, 1 University Avenue, Aglantzia, Nicosia 2109, Cyprus; chrysostomou.c.andreas@ucy.ac.cy

**Keywords:** human papillomavirus, cervical cancer, HPV testing

## Abstract

The human papillomavirus is one of the most common sexually transmitted viruses, and an infection from this virus may become persistent, leading to diseases such as cervical cancer. In the past, cytology-based methods such as the Papanicolaou (Pap) test were imperative to identify the disease at a stage where it can be treated. However, since the 1980s where the etiological association of HPV and cervical cancer was identified, new tests began emerging directed towards identifying the virus. Furthermore, as the biology of HPV along with the relationships with its host are elucidated, these tests and treatments further advance. Recently in Europe, there is a movement towards the implementation of HPV testing methodologies in national screening programs to precede cytological testing. These screening strategies are recommended by the European guidelines and the World Health Organization. This review presents the current HPV testing methodologies, their application in organized population-based cervical cancer screening programs based on the most recent European guidelines, and their implementation status in countries in Europe.

## 1. Introduction

The human papillomavirus (HPV) is a mucotropic or cutaneotropic, double-stranded, non-enveloped virus. The genome is approximately 8 kbp long and it encodes for early regulatory proteins (E1, E2, E4, E5, E6, and E7), late structural proteins (L1 and L2), as well as the long control region (LCR) [1,2]. HPV is primarily a sexually transmitted virus, and more than 80% of sexually active people are infected by this virus during their life [3]. An infection caused by HPV is usually transient and asymptomatic, and it is cleared by the host’s immune system in six months to two years [4]. In the case where an HPV infection becomes persistent, it may lead to pre-cancer, which may progress to cancer if it is not identified early enough to be treated [5]. However, it is important to note that the majority of HPV genotypes are not highly carcinogenic [6]. In fact, there are currently 226 HPV genotypes and new HPVs that are continuously being discovered [7]. These HPV genotypes are classified in accordance with carcinogenicity as denoted by the International Agency for Research on Cancer (IARC-WHO). The low risk (lr) HPVs include the genotypes 6 and 11, which are not associated with carcinogenicity, mainly causing anogenital warts and oral papillomas [8,9]. The high risk (hr) carcinogenic HPV types (Group 1) are 16, 18, 31, 33, 35, 39, 45, 51, 52, 56, 58, 59, and HPV68 classified as probably carcinogenic (Group 2A), and HPV types 26, 30, 34, 53, 66, 67, 69, 70, 73, 82, 85, and 97 that are classified as possibly carcinogenic (Group 2B) [9]. HrHPVs lead to cancers such as cervical, vulvar, vaginal, anal, penile, head and neck, oral cavity, and larynx [2,10].

As it is evident, HPV is associated with numerous cancers, as well as cervical cancer, which is the fourth most common cause of cancer incidence and mortality in women, with an estimated worldwide incidence of 570,000 cases and 311,000 deaths in 2018 [11]. Although the incidence and mortality of cervical cancer have decreased since the development of the Pap test in the 1940s [11,12] and the implementation of cervical cancer screening programs in the 1960s–70s [13], the disease remains a serious public health concern [11]. With the discovery of the etiological association of HPV and cervical cancer in 1983 [14], new methods were developed for cervical cancer screening by designing tests that focus on identifying the virus/infection rather than the onset of disease.

In light of the recent movement towards population-based primary HPV screening [15], this review summarizes the current methodologies and strategies of HPV testing employed for the screening of HPV infection and cervical cancer.

## 2. HPV Testing: Methodologies and Implementation in Screening Programs

### 2.1. Cervical Cytology and Reasons That Lead to HPV-Based Approaches

Methods that are based on cytology such as the Pap test rely on the morphologic interpretation of cells collected from the woman’s cervix in order to identify if there is any degree of cellular degeneration [16]. Cytology based testing has been the gold standard to test for cervical cancer since the development of the Pap test, primarily due to its high specificity; however, it is characterized by certain drawbacks. It has poor reproducibility, and it can be affected by blood and mucus obscuration, imperfect fixation, and non-uniform distribution of cells. These issues may hinder the already difficult interpretation of results; hence, highly trained personnel are required [17,18]. Furthermore, despite alternatives and efforts to improve upon methods relying on cytology, such as the UltraFast staining technique [19], liquid-based cytology (LBC) with the ThinPrep^®^ Pap test (Hologic, Inc, Marlborough, MA, USA) and SurePathTM (SP; BD Diagnostics, Burlington, NC, USA) [18,20], and visual inspection by acetic acid or Lugol’s iodine [21], the sensitivity is not optimal, yielding uncertain results, such as atypical squamous cells of undetermined significance (ASCUS, or ASC-US after the 2001 Bethesda Workshop). These results require close and constant follow up, which may lead to increased referrals for colposcopy and treatment [22].

### 2.2. Reasoning of HPV Testing Implementation in Screening Programs

HPV testing is a highly sensitive, objective molecular approach to screen for cervical cancer that does not rely on the morphologic interpretation of results, which in cytology may be subject to inter-observer variability [23]. HPV testing relies on the detection of the virus or effects of the viral infection to discover high-grade cervical dysplasia [24]. A benefit of HPV testing is that it allows for longer screening intervals due to fact that hrHPV requires a longer duration of time to progress to cancer than cells that are in the pre-cancer stage [25]. In fact, the European guidelines recommend that primary HPV testing may be performed at a five-year interval with the possibility to be extended to up to 10 years based on the medical history and age of the woman [26,27]. Furthermore, along with high clinical sensitivity and objectiveness, HPV testing also has a high negative predictive value (NPV), low training requirements, high reproducibility, and a high throughput capacity [28,29,30]. When taken together, and in conjunction with HPV vaccination, primary HPV testing every five years with cytology as a triage proved to be a more cost-effective option [31]. However, it is important to take in account the biology of the virus in relation to its host in order to decide the starting screening age. Thus, to account for the relatively lower specificity of the test and to avoid unnecessary follow-up or overtreatment of women likely having transient HPV infections, the European guidelines recommend the starting age for primary HPV testing to be after the age of 30 and up to 35 [26,27]. Yet, in countries or regions where a primary cytology program is predominant and successful, the European recommendations allow the program to continue to run for the ages 20–30, while implementing primary HPV testing for ages above 30 [26,27]. Conversely, the age to exist a screening program is recommended to be 60–65, although women with a negative HPV screening history from the age of 55 are at low risk for an HPV infection that may become persistent and subsequently develop to cervical cancer [27,32]. Additionally, cytology testing has also been reported as suboptimal for women of this age range and for post-menopausal women due to epithelial atrophy and less accessible transformation zones, which are found in the cervical canal [33,34]. Nonetheless, since the risk still exists for that cohort, the age to stop screening with HPV testing is still under consideration, and it is continuously revised as scientific evidence is accumulated [33].

### 2.3. HPV Testing Assays and Validation

A concern of screening programs, particularly those based on HPV testing, stems from the fact that many viral targets (e.g., E6/E7 HPV mRNA, or L1, L2, E6/E7 HPV DNA, whole genome HPV DNA) may be used to detect an HPV infection. Due to this aspect of HPV testing, there is a plethora of tests available either in-house or commercial, yet only a number of them have been validated and approved for routine testing. Currently, there are 254 distinct commercial tests, and more than 425 variants of those tests have been identified [35]. These tests can be divided into hrHPV DNA, hrHPV with partial genotyping for the main hrHPVs, full HPV DNA genotyping tests, HPV DNA type/group-specific tests, hrHPV E6/E7 mRNA tests, in situ hybridization DNA in mRNA-based HPV tests, as well as tests identifying HPV DNA targeting miscellaneous HPV types [35]. These tests are based on the principles of Polymerase Chain Reaction (PCR) amplification coupled with sequencing, restriction fragment length polymorphism (RFLP) analysis, or hybridization assays. Additionally, other tests are based on real-time detection, transcription-mediated amplification (TMA) or nucleic-acid sequenced based amplification (NASBA) [36]. Namely, HPV tests that are currently circulating are Xpert HPV (Cepheid), PapilloCheck (Greiner Bio-One), INNO-LiPA HPV Genotyping Extra (Innogenetics), Cobas 6800/8800 HPV Test (Roche Molecular Systems Inc., Alameda, CA, USA), and HPV-Risk Assay (Self-screen BV, Amsterdam, Netherlands); hence, proper criteria (Meijer Criteria) and validation initiatives are required to ascertain which assays are appropriate for cervical cancer screening [35,37,38,39]. Specifically, an international expert committee in 2009 proposed criteria to denote assays suitable for cervical cancer screening [37,39]. These criteria aim to assure that candidate hrHPV tests should have an ideal balance between clinical specificity and sensitivity for the detection of CIN2/3, consequently reducing the number of follow up tests a woman has to undergo. For these purposes, new hrHPV DNA assays are compared to the Hybrid Capture 2 (HC2) or GP5+/6+ PCR- enzyme immunoassay (EIA) tests that are used as comparator tests due to their extensive clinical validation. Furthermore, each new test should be highly reproducible and applied to a clinically relevant set of samples characterized by various degrees of CIN from a screening cohort of women within the 30–60-year age group [37,39]. In this effort of a standardized validation, the international framework “Validation of HPV Genotyping Tests” (VALGENT) was launched in order to provide a comprehensive validation and comparison for HPV genotyping tests to be used for clinically relevant results, which is achieved through the employment of sample populations that are relevant for primary cervical cancer screening [40]. As of July 2019, there are 15 commercial HPV assays that are either completely or partially validated to be used for cervical cancer diagnostics based on primary HPV testing [40,41,42]. The list includes but is not limited to HC2, HPV DNA Test (Qiagen), cobas 4800 HPV Test (Roche), APTIMA HPV Assay (Hologic), and BD Onclarity HPV Assay (Becton Dickinson) [41]. In Table 1, a selection of HPV tests is presented that are used in primary HPV screening and triage testing, as well as tests used as comparator tests for validation purposes, indicating their technical characteristics, the category they are assigned to, their validation, and intended use [24,35,38,43,44,45,46,47,48,49,50,51,52,53].

### 2.4. Screening Algorithms Employing Primary HPV Testing

**Figure 1:**
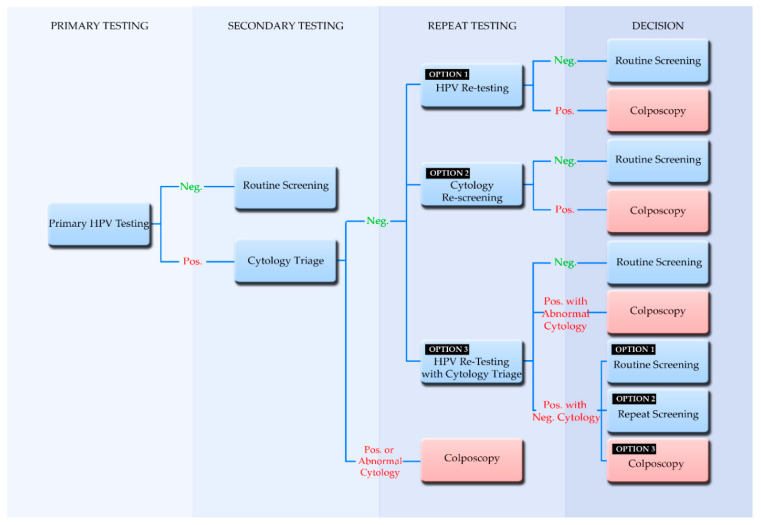
Management algorithm in primary HPV screening. Abnormal cytology refers to a borderline or more severe cytological result. This figure was adapted from Chrysostomou et al. (2018) [15]. This algorithm was developed based on “The supplements of the second edition of the European Guidelines for Quality Assurance in Cervical Cancer Screening of 2015” [27].

It is important to note that primary HPV testing is optimally part of a screening algorithm that employs triage and follow-up testing. This screening algorithm is imperative for the proper management of test results. Thus, with the expected increase in positive results from HPV testing, the European guidelines recommend cytology testing as a triage in order to avoid a large influx of referrals for colposcopy [27]. An HPV-based screening algorithm begins with the primary test as shown in Figure 1, where a positive HPV result moves further along the algorithm to secondary testing and cytology triage. In the case that the primary HPV test has genotyping capabilities and it is positive for HPV16 and HPV18, then it is acceptable for the woman to be directly referred for colposcopy, even without a cytology intermediate test [27]. If cytology triage testing shows a positive result then it is referred for colposcopy. A benefit of primary HPV testing followed by cytology triage is that HPV negative results, which may have had the possibility to be ASC-US cases, would not be referred to and burden cytological testing, since they are essentially unlikely to pose the threat of pre-cancer or cancer [54]. Additionally, knowledge of the HPV status has been associated with an increase in the predictive value of the cytologist [30]. This still leaves the matter of HPV-positive, cytology-negative women (repeat testing in Figure 1) who are still at risk for having been identified with hrHPV. The European guidelines call for shorter intervals of repeat testing; however, evidence is still inconclusive to suggest one specific route [27]. For this reason, three possible routes are suggested for policy makers, where repeat testing may be performed through HPV testing, cytology, or HPV testing with cytology triage. Ultimately, positive results of hrHPV and abnormal cytology are referred to colposcopy (Decision, Figure 1) and in the case where high-grade cervical lesions are diagnosed, they are followed by treatments such as surgical excision, cryotherapy, and the loop electrosurgical excision procedure (LEEP) [55]. Despite the high success rate of these treatments, there is still a chance for residual or recurrent pre-cancer, and for this reason, HPV testing is also suggested for post treatment monitoring [56,57].

### 2.5. Participation in Screening and the Implementation of Self-Sampling

In the implementation of any methodology in a screening program, participation is imperative for its success. In order to tackle this issue, which may be caused by women having difficulties in accessing health services, self-sampling is also considered as an option [58]. In this regard, it is also important to consider the attitude of women towards self-sampling. In a study by Leinonen et al. (2018), high acceptability and positive attitudes were observed towards self-sampling, with no differences in preference based on age, education, and marital status [59]. Additionally, even though women expressed more confidence in samples taken from trained personnel they would still prefer self-collection at home [59]. Yet, self-sampling can also be performed at a specialized facility, by the women themselves or with trained personnel assistance, thereby providing the option to ask questions and receive assurance that the sample was taken correctly [60]. Currently, kits for hrHPV self-sampling show great promise as means to increase participation in screening programs and they can achieve a higher degree of accuracy than those for cytology, reportedly having similar sensitivity and specificity to samples taken by trained medical personnel [58,59]. Importantly, in a meta-analysis study by Arbyn et al. (2018), hrHPV testing from self-sampling was shown to have comparable sensitivity to detect cervical intraepithelial neoplasia (CIN2+) and CIN3+, with almost as much specificity in comparison to clinical samples [61]. Interestingly, PCR-based hrHPV testing from self-sampling was shown to have higher sensitivity and specificity (to exclude CIN2+) than signal amplification-based techniques, while mRNA testing and hrHPV DNA testing from self-sampling showed similar specificity but lower sensitivity than clinically collected samples [61].

### 2.6. Implementation of Primary HPV Testing in Europe

**Figure 2:**
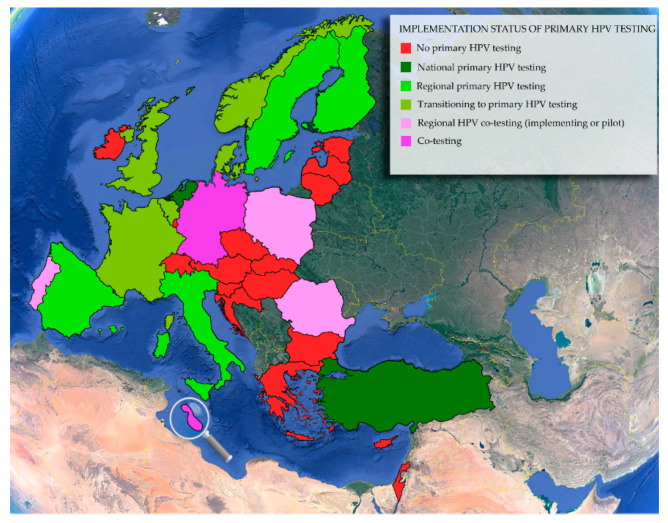
The implementation status of primary HPV testing in E.U. member states and some E.U. associated countries. This figure was adapted from Chrysostomou et al. (2018) [15], and it is updated with new information. The magnifying glass serves to enlarge the island of Malta. It is important to state that this is a rapidly changing field and that the status of implementation could not be confirmed for all countries from two independent sources.

Regardless of the HPV test the policy makers may choose to use within population-based cervical cancer screening programs, it has to be performed in qualified accredited laboratories and has to follow international standards [27,42]. In Europe, there is a recent movement towards primary HPV testing, with countries having implemented such a program or being in the process of implementation, as shown in Figure 2. Norway, the United Kingdom, The Netherlands, Germany, Belgium, France, Denmark, Malta, and Turkey have decided to adopt primary HPV testing [15,42,62,63,64]. While Turkey and The Netherlands have already implemented a national program with primary HPV testing, other countries are still underway to assimilate HPV testing as a primary method in their screening programs [15,42]. It is also important to note that Germany has opted to perform co-testing for women above the age of 35 [65], and Malta may also include HPV testing for women above the age of 30 [66]. With an increasing number of countries in Europe (Figure 2) and around the world, such as Australia [67] and Singapore [68], implementing HPV-based screening, the evidence regarding both the implementation process and HPV testing is growing, serving as a basis for the universal transition towards organized population-based primary HPV testing.

## 3. Emerging Diagnostic Methods

New cervical cancer diagnostic methods are constantly being developed, which do not necessarily identify HPV targets, and could be used for triage. These methods include testing for DNA methylation, host factors (biomarkers that arise from cellular modifications induced by the viral infection), and artificial intelligence-assisted cytology [69,70,71,72]. DNA methylation tests are based on aberrant methylation of cellular DNA, which are associated with a number of diseases, and cancers are no exception. For cervical cancer, such markers include the 19 (chemokine (C–C)-motif)-like) member A4 (FAM19A4) and microRNA 124-2 (miR124-2) genes [73]. Although this method is still improving, it is showing significant promise due to its objectivity and capabilities for risk stratification [70,73]. In fact, DNA methylation tests show higher specificity than cytology for ASC-US cases and higher sensitivity than tests relying on HPV16/18 genotyping, constituting important candidates for triage tests [74]. Conversely, certain host factors such as p16^INK4a^–Ki-67 are upregulated as a result of the overexpression of the E6/E7 viral oncogenes [69]. These markers are identified through staining techniques and have been found to have virtually 100% sensitivity for CIN3+ compared with CIN2+ when combined with high-grade cytology, as well as having comparable results to routine LBC and HPV testing. It is suggested that p16^INK4a^–Ki-67 staining techniques could be used as an efficient tool for triage to reduce referrals to colposcopy [69,75]. Another promising method to be used for triage is based on artificial intelligence. This technique relies on deep learning algorithms that have been developed in order to deal with a large amount data, to segment cytoplasm, and detect cervical dysplasia [72]. Even though cytological readings assisted by artificial intelligence have been shown to have similar accuracy as cytological analyses performed by capable cytologists, they are still in the early stages and require further investigation before they can be reliably used in screening programs [72,76]. Similarly, with DNA methylation and biomarker detection, large-scale and long-term prospective studies are required to clarify their role in screening for cervical cancer.

## 4. Conclusions

An increasing number of countries is opting to implement HPV testing in their screening programs realizing the benefits this methodology has to offer as a primary method of screening [15,42]. It is understandable that such a shift demands political commitment and a great deal of system and infrastructure restructuring, since the majority of countries started out with cytology-based testing. However, cytology-based programs may be costly and inefficient, thereby threatening the public health budget and straining the population being tested [12,17]. When primary HPV testing is part of an organized population-based screening program, it offers a more cost efficient, accurate, sensitive method that provides a longer period of “peace of mind” to the women who are part of the program [15,24,31]. Moreover, with the recently launched international framework VALGENT [40], each country opting to follow the European and WHO recommendations to implement HPV testing [27,30] is able to reliably select validated, high quality HPV tests, which, when coupled with an organized, population-based program that has high coverage, will effectively reduce the burden of cervical cancer.

## Figures and Tables

**Table 1:** Selection of tests that use different targets and methodologies for HPV detection used in HPV screening as well as tests used as comparator tests for validation purposes.

Tests	Hybrid Capture 2 (Qiagen)	GP5+/6+ EIA ^a^	Cobas 4800 HPV Test (Roche)	APTIMA HPV Assay (Hologic)	BD Onclarity HPV Assay
Type of assay	Signal amplification, hybrid capture	PCR, probe hybridization	Real-time PCR detection	Transcription mediated amplification, probe hybridization	Real-time PCR detection
Targets	DNA, Whole viral genome	L1 DNA, 150 bp	L1 DNA 200 bp	E6/E7 mRNA	E6 and E7 DNA
HPV Subtypes detected	16, 18, 31, 33, 35, 39, 45, 51, 52, 56, 58, 59 and 68	16, 18, 31, 33, 35, 39, 45, 51, 52, 56, 58, 59, 66, and 68	16, 18, 31, 33, 35, 39, 45, 51, 52, 56, 58, 59, 66 and 68. Individual genotyping for: 16, 18	16, 18, 31, 33, 35, 39, 45, 51, 52, 56, 58, 59, 66, and 68. Reflex Partial genotyping for: 16, 18–45	33–58; 56–59–66; 35–39–68 ^f^. Individual genotyping for: 16, 18, 31, 45, 51, and 52
Internal Controls Human genes	NO	NO	Internal human β-globin control	Internal RNA transcript (HPV16 E6/7) control	Internal human β-globin control
Capacity Batch size	88	96 samples in 9.5 h ^e^	96	Panther system 100 and 250 test /Tigris DTS system 250	46
VALGENT Validation	Standard comparator tests for validation ^b^	Standard comparator tests for validation ^b^	YES	YES	YES
US FDA ^c^ Validation	YES	NO	YES	YES	YES
CE Mark ^d^ Validation	YES	YES	YES	YES	YES
Uses within a screening program	ASC-US Triage, test-of-cure	ASC-US Triage, test-of-cure	ASC-US Triage/co-testing/Primary testing	ASC-US Triage/co-testing	ASC-US Triage/co-testing/Primary testing

^a^ GP5+/6+ enzyme immunoassay (EIA), DDL Diagnostic Laboratory (Rijswijk, The Netherlands). ^b^ HC2 and GP5+/6+ PCR-EIA are extensively clinically validated in randomised trials, used as standard comparator tests for HPV assay validation. ^c^ United States Food and Drug Administration. ^d^ European Commission CE (Conformité Européenne) marking. ^e^ For the GP5+/6+ enzyme immunoassay (EIA), the number of tests in the kit was reported along with the time required for results [44]. ^f^ The BD Onclarity HPV Assay genotypes eight genotypes in three groupings (HPV 33 and 58; HPV 56, 59, and 66; and HPV 35, 39, and 68).

## Abbreviations

HPV	Human papillomavirus
Pap test	Papanicolaou test
Lr	Lower risk
Hr	High-risk
ASC-US	Atypical squamous cells of undetermined significance
NPV	Negative predictive value
LEEP	Loop electrosurgical excision procedure
RFLP	Restriction fragment length polymorphism
TMA	Transcription mediated amplification
NASBA	Nucleic-acid sequenced based amplification
VALGENT	Validation of HPV Genotyping Tests
HC2	Hybrid Capture 2
EIA	Enzyme Immunoassay
US FDA	United States Food and Drug Administration
LMNX	Luminex
IARC	International Agency for Research on Cancer
LBC	Liquid-Based Cytology
PCR	Polymerase Chain Reaction
